# Retrospective registry of patients with locally advanced/metastatic HR^+^/HER2^−^ breast cancer treated in clinical practice in Andalusia

**DOI:** 10.1007/s12094-024-03510-8

**Published:** 2024-06-03

**Authors:** Natalia Chavarría Piudo, Isabel Blancas, Encarna González Flores, Fernando Henao Carrasco, Pilar López Álvarez, David Morales Pancorbo, Salvador Gámez Casado, María de la Cabeza Lomas Garrido, José Manuel Rodríguez García, Antonia Martínez Guisado, Adrián Sánchez Vega, Manuel Ruíz Borrego

**Affiliations:** 1 Medical Oncology Service, Instituto de Investigación E Innovación Biomédica de Cádiz (INIBiCA), Institute for Biomedica Research and Innovation, Hospital Universitario de Jerez de La Frontera, Jerez, Cadiz, Spain; 2 Medical Oncology Service, Instituto de Investigación Biosanitaria de Granada (Ibs.Granada) and Medicine Department, Hospital Universitario San Cecilio, Granada University, Granada, Spain; 3 Medical Oncology Service, Hospital Universitario Virgen de Las Nieves, Instituto de Investigación Biosanitaria de Granada (Ibs. GRANADA), Granada, Spain; 4 Medical Oncology Service, Hospital Universitario Virgen Macarena, Fundacion Para La Gestión de La Investigacion en Salud de Sevilla (FISEVI), Seville, Spain; 5 Medical Oncology Service, Hospital Universitario Virgen de Valme, Seville, Spain; 6 Medical Oncology Service, Hospital Universitario Juan Ramón Jiménez, Huelva, Spain; 7 Medical Oncology Service, Instituto de Investigación E Innovación Biomédica de Cádiz (INIBiCA), Institute for Biomedica Research and Innovation, Hospital Universitario Puerta del Mar, Cádiz, Spain; 8 Medical Oncology Service, Hospital Universitario de Jaén, Jaén, Spain; 9 Medical Oncology Service, Hospital Punta de Europa, Álgeciras, Cádiz, Spain; 10 Medical Oncology Service, Hospital Universitario de Torrecárdenas, Almería, Spain; 11 Medical Oncology Service, Hospital Universitario de Puerto Real, Instituto de Investigación e Innovación Biomédica de Cádiz (INIBiCA) [Institute for Biomedica Research and Innovation], Puerto Real, Cádiz, Spain; 12 Medical Oncology Service, Hospital Universitario Virgen del Rocío, Fundacion para la Gestión de la Investigacion en Salud de Sevilla (FISEVI), Sevilla, Spain

**Keywords:** Cyclin-dependent kinase 4/6 inhibitors, Breast cancer, Palbociclib, Ribociclib, Metastatic breast cancer, Real-world evidence

## Abstract

**Background:**

Limited data are available regarding the real-world effectiveness and safety of Cyclin Dependent Kinase 4/6 inhibitor (CDK4/6i) (palbociclib/ribociclib) just as a first-line treatment for patients with hormone receptor-positive/human epidermal growth factor receptor 2-negative (HR + /HER2‒) metastatic breast cancer (MBC).

**Objective:**

To assess whether clinical or demographic characteristics limit access to first-line CDK4/6i treatment in clinical practice in the Autonomous Community of Andalusia (Spain) between November 2017 and April 2020. In addition, effectiveness will be described in an exploratory analysis.

**Methods:**

Physicians from 12 centers participated in selecting demographic and clinical characteristics, treatment, and outcome data from women with HR + /HER2- MBC treated with or without CDK4/6i in addition to hormonal in the first-line setting, in a 3:1 proportion. Kaplan–Meier analysis estimated progression-free rates (PFRs) and survival rates (SRs).

**Results:**

A total of 212 patients were included, of whom 175 (82.5%) were in the CDK4/6i treatment group and 37 (17.5%) were in the non-CDK4/6i treatment group (control group). Patients in the CDK 4/6i treatment group were younger (p = 0.0011), the biopsies of the metastatic site at the moment of the relapse were most commonly performed (p = 0.0454), and had multiple metastatic sites (p = 0.0025). The clinical benefit rate (CBR) was 82.3% in the CDK4/6i group and 67.8% in the control group. Median time to a progression event or death (PFS) was 20.4 months (95%CI 15.6–28) in the CDK4/6i group and 12.1 months (95%CI 7.9–not reached) in the control group.

**Conclusions:**

Younger patients, biopsies of metastatic disease and with multiple metastatic sites were more frequently treated with CDK4/6i in our daily clinical practice.

**Supplementary Information:**

The online version contains supplementary material available at 10.1007/s12094-024-03510-8.

## Introduction

Breast cancer (BC) is the most common cancer that affects women, accounting for almost one-third of all cancer cases [1]. In 2020, globally, 2.3 million women were diagnosed with BC, 685,000 deaths were reported, and 7.8 million women who had been diagnosed with breast cancer in the past five years were still alive, making it the world's most prevalent cancer [2]. In Spain, 34,088 new cases of BC were diagnosed in the same year [3]. Approximately 6% of the new BC diagnosis are metastatic breast cancer (MBC), with a low 5-year survival rate of only 29% [4]. The most common subtype of breast cancer is hormone receptor-positive/human epidermal growth factor receptor 2-negative (HR + /HER2−), accounting for 68% of cases [4].

Chemotherapy, hormone therapy, and targeted therapy are treatment options for HR + /HER2− MBC [5]. In recent years, the emergence of cyclin-dependent kinase 4/6 inhibitors (CDK4/6i) has provided another option of treatment for these patients. These inhibitors induce cell cycle arrest, inhibiting cancer cells´ proliferation. Currently, three drugs are available (palbociclib, ribociclib, and abemaciclib) that have been combined with endocrine and targeted therapies to treat HR + /HER2− MBC, with palbociclib being the first approved for this indication in 2017 [5]. To assess the effectiveness and risks of these drugs, in addition to pivotal clinical trials, real-world evidence (RWE) studies have provided additional data that not only confirm the clinical trial results but also give valid information in their results in clinical practice according to the population treated [6–10].

A recent systematic review showed that Palbociclib was the most chosen treatment in real-world studies of CDK4/6i for HR + /HER2− MBC [6]. CDK4/6i are safe and effective treatments for HR + /HER2− MBC in routine practice. However, fewer data are available regarding the real-world effectiveness and safety of ribociclib and abemaciclib [6]. Real-life studies have intrinsic biases, such as confusion, missing data, reporting, misclassification, and treatment choice, but these can be reduced with statistical adjustments. The choice or selection of treatment is often influenced by the baseline characteristics of the patient; if the two groups are compared, the effect may be attributed to differences in treatment efficacy, when in fact they are due to the differential characteristics of the patients [11–13]. To overcome these limitations, several studies included a large number of patients and used statistical methodology to homogenize the patient population included in real-life studies [14, 15].

Finally, the demographic and clinical heterogeneity of patients in RWE studies may lead to biases in the comparison of treatments [11–13]. There may even be more important confounding factors, such as administrative, logistical, or insurance coverage-based barriers [16, 17].

Therefore, there is a need to perform studies in real-world clinical settings of CDK4/6i. The objective of this study was to compare the clinical and demographic characteristics of HR + /HER2 − MBC patients receiving first-line CDK4/6i (palbociclib/ribociclib) and those without CDK4/6i treatment under the usual circumstances of healthcare in Andalusia (palbociclib/ribociclib). In addition, an exploratory analysis of the effectiveness and safety data of both cohorts will be analyzed.

## Materials and methods

### Study design

This was a multicenter, retrospective, observational, cohort analysis of patients with HR + /HER2 − metastatic breast cancer who were treated in clinical practice in the Autonomous Community of Andalusia. The study protocol was approved by the Ethics Committee of the Hospital Virgen Macarena—Virgen del Rocio of Sevilla on July 28, 2020.

### Study population

All patients aged 18 years or older with confirmed HR + /HER2 − metastatic breast cancer were treated according to usual clinical practice in public centers in Andalusia, who started treatment first line with CDK4/6i (palbociclib or ribociclib), and those without CDK4/6i between November 1, 2017 and April 30, 2020, were included in the study. Patients treated in the context of a clinical trial were excluded from the study.

### Data source and variables

Patients were identified from 12 centers from the Andalusian Public Health System -Junta de Andalucía, Spain. The following information was extracted from the review of electronic health records (EHR): demographic and clinical characteristics, tumor and advanced disease characteristics, and treatments received. Two cohorts were defined based on CDK4/6i prescription (Yes/No) in a 3:1 proportion.

### Outcomes

The primary endpoint was the comparison of the clinical and demographic characteristics (age, sex, menopausal status, ECOG, and area of residence) between patients treated with first-line CDK4/6 inhibitors (palbociclib or ribociclib) and those not treated with CDK4/6 inhibitors. Secondary outcomes included population characteristics based on treatment with or without a CDK4/6 inhibitor, population by metastatic location (visceral, non-visceral, and bone-exclusive), population by DFI (disease-free survival) (newly metastatic disease, ≤ 12 months and > 12 months), treatment duration, percentage of patients with dose reduction, percentage of patients with dose interruption, percentage of patients with permanent withdrawal, or the result based on the rate of objective response, and CBR (defined as the total number (or percentage) of patients who achieved a complete response, partial response, or had stable disease for 6 months or more) for the line of treatment were also assessed. Progression-free survival (PFS) was defined as the time from first-line treatment initiation to disease progression or death. Progression-free survival 2 (PFS2) was defined as the time from second-line treatment initiation to disease progression or death. Additional safety outcomes assessed were the percentage of patients with febrile neutropenia, G3/4 fatigue, neutropenia, AST/ALT elevation, and QTc prolongation during treatment with palbociclib and ribociclib. AEs were graded for severity according to the National Cancer Institute Common Terminology Criteria for Adverse Events version 4.0 and classified according to Medical Dictionary for Regulatory Activities (MedDRA, v20.0).

### Statistical analysis

Descriptive statistics were used to summarize patient demographics, tumor characteristics, and treatment characteristics. Continuous variables were summarized as mean and standard deviation (SD), and median and interquartile range (IQR), as required. Categorical variables were described as frequencies and percentages. Variables were transformed and the results were stratified according to data distribution. Differences were compared using the chi-square test for categorical variables, and nonparametric tests for continuous variables. Kaplan–Meier survival analysis was used to determine median time to a progression event, defined as disease progression, start of the next line of treatment, or death. Statistical significance was set at p < 0.05. All statistical analyses were performed using the SAS statistical software version 9.3 (SAS Institute Inc., Cary, NC, USA).

## Results

### Patient characteristics

Between November 1, 2017 and April 30, 2020, a total of 273 patients were screened (Figure S1 in the Supplementary Appendix includes a flowchart of study patients). The final analysis included 212 patients, of which 175 (82.5%) were in the CDK4/6i treatment group and 37 (17.5%) in the non-CDK4/6i treatment group (control group). Most of the patients included in the study were female (n = 211, 99.5%). The median age was 58 years (IQR: 51–69) and 151 (71.2%) patients were postmenopausal women. Differences were observed between the two treatment groups (Table 1). In this regard, CDK 4/6i-treated patients were younger (*p* = 0.0011), and biopsies of metastatic sites were more frequent in the CDK 4/6i group than in the control group (*p* = 0.0454). The control group hadwerewe more oligo-metastatic patients when compared the CDK 4/6 inhibitor group, and the difference was statistically significant (*p* = 0.0025), and differences between the to comparing groups were found in relation to the time of diagnosis of metastatic disease (*p* = 0.0304).

**Table 1:** Baseline Demographic and Clinical Characteristics of patients (*n* = 212)

	CDK 4/6i	Control	Total	*p* value
Age (years), *n*	175	37	212	0.0011
Mean (SD)	58.1 (11.8)	67.6 (15.8)	59.7 (13.0)	
Sex, *n*	175	37	212	NS
Male, *n* (%)	1 (0.6)	0 (0)	1 (0.5)	
Female, *n* (%)	174 (99.4)	37 (100)	211 (99.5)	
Menopause, *n*	175	37	212	NS
Yes, *n* (%)	123 (70.3)	28 (75.7)	151 (71.2)	
No, *n* (%)	51 (29.1)	9 (24.3)	60 (28.3)	
Does not apply, *n* (%)	1 (0.6)	0 (0)	1 (0.5)	
Living area, *n*	175	37	212	NS
Rural, *n* (%)	86 (49.1)	16 (43.2)	102 (48.1)	
No rural, *n* (%)	89 (50.9)	21 (56.8)	110 (51.9)	
ECOG, *n*	139	24	163	NS
0, *n* (%)	109 (78.4)	19 (79.2)	128 (78.5)	
1, *n* (%)	22 (15.8)	3 (12.5)	25 (15.3)	
2, *n* (%)	6 (4.3)	2 (8.3)	8 (4.9)	
3, *n* (%)	2 (1.4)	0 (0)	2 (1.2)	
Diagnosis of metastatic disease moment, *n*	175	37	212	0.0304
De novo, *n* (%)	36 (20.6)	13 (35.1)	49 (23.1)	
≤ 5 years, *n* (%)	57 (32.6)	15 (40.5)	72 (34.0)	
> 5 years, *n* (%)	82 (46.9)	9 (24.3)	91 (42.8)	
Time on hormone therapy prior to diagnosis of metastatic disease, *n*	121	20	141	NS
≤ 24 months, *n* (%)	20 (16.5)	5 (25.0)	25 (17.7)	
> 24 months, *n* (%)	101 (83.5)	15 (75.0)	116 (82.3)	
Metastasis, *n*	175	37	212	0.0025
Single, *n* (%)	33 (18.9)	16 (43.2)	49 (23.1)	
Multiple, *n* (%)	142 (81.1)	21 (56.8)	163 (76.9)	
Metastasis location, *n*	175	37	212	
Pulmonary, *n* (%)	56 (32.0)	7 (18.9)	63 (29.7)	NS
Hepatic, *n* (%)	41 (23.4)	7 (18.9)	48 (22.6)	NS
Bone, *n* (%)	123 (70.3)	22 (59.5)	145 (68.4)	NS
Other, *n* (%)	58 (33.1)	12 (32.4)	70 (33.0)	NS
Progression during adjuvant chemotherapy, *n*	72	9	81	NS
Yes, *n* (%)	4 (5.6)	1 (11.1)	5 (6.2)	
No, *n* (%)	68 (94.4)	8 (88.9)	76 (93.8)	
Progression during adjuvant hormonal therapy, *n*	127	23	150	NS
Yes, *n* (%)	70 (55.1)	14 (60.9)	66 (44.0)	
No, *n* (%)	57 (44.9)	9 (39.1)	84 (56.0)	
Biopsy	175	37	212	0.0454
Yes, *n* (%)	91 (52.0)	12 (32.4)	103 (48.6)	
No, *n* (%)	84 (48.0)	25 (67.6)	109 (51.4)	
Progesterone receptors in metastatic diagnosis, *n*	91	12	103	NS
Positive, *n* (%)	55 (60.4)	7 (58.3)	62 (60.2)	
Negative, *n* (%)	21 (23.1)	4 (33.3)	25 (24.3)	
Unknown, *n* (%)	15 (16.5)	1 (8.3)	16 (15.5)	
Estrogen receptors in metastatic diagnosis, *n*	91	12	103	NS
Positive, *n* (%)	83 (91.2)	12 (100)	95 (92.2)	
Negative, *n* (%)	3 (3.3)	0 (0)	3 (2.9)	
Unknown, *n* (%)	5 (5.5)	0 (0)	5 (4.9)	
Proliferative tumor grade at metastatic diagnosis, *n*	91	12	103	NS
Grade 1, *n* (%)	3 (3.3)	1 (8.3)	4 (3.9)	
Grade 2, *n* (%)	8 (8.8)	2 (16.7)	10 (9.7)	
Grade 3, *n* (%)	12 (13.2)	1 (8.3)	13 (12.6)	
Unknown, *n* (%)	68 (74.7)	8 (66.7)	76 (73.8)	
Ki67 expression rate, *n*	56	8	64	NS
Ki67 ≤ 20, *n* (%)	29 (51.8)	5 (62.5)	34 (53.1)	
Ki67 > 20, *n* (%)	27 (48.2)	3 (37.5)	30 (46.9)	
Treatment lines, *n*	175	37	212	NS
1st line of treatment, *n* (%)	114 (65.1)	18 (48.6)	132 (62.3)	
2nd line of treatment, *n* (%)	29 (16.6)	9 (24.3)	38 (17.9)	
3rd line of treatment,* n* (%)	32 (18.3)	10 (27.0)	42 (19.8)	

Overall, 151 (86.3%) patients were treated with palbociclib and 21 (12.0%) with ribociclib while, 3 (1.7%) patients started with ribociclib and switched to palbociclib.

### Effectiveness outcomes

#### Overall response

The CBR was 82.3% in first-line CDK4/6i-treated patients (164 patients) for metastatic disease vs 67.8% control (28 patients) (OR 2.2 (0.9–5.3); *p* = 0.076). The disease progression rate was lower in the CDK4/6i group (17.7%) than that in the control group (32.1%) (Table 2).

**Table 2:** Best overall response by group

	CDK 4/6i (*n* = 175)	Control (*n* = 37)
	1st line^a^	1st line^b^
Response *n* (%)		
Complete response (CR)	14 (8.5)	2 (7.1)
Partial response (PR)	48 (29.3)	9 (32.1)
Stable disease (SD)	73 (44.5)	8 (28.6)
No response, progression of disease	29 (17.7)	9 (32.1)
Clinical Benefit Rate (CR + PR + SD)	135 (82.3)	19 (67.8)
Total	164 (100)	28 (100)

^a^ Data for better response are not available for 7 patients and in 4 patients the response was not evaluable

^b^ Data for better response are not available for 2 patients and in 7 patients the response was not evaluable

#### Progression-free survival and overall survival

During first-line treatment, 22 patients (59.5%) in the control group had experienced a progression event, and 15 patients (40.5%) were censored without a progression event. In the CDK4/6i group, 84 patients (48%) experienced disease progression, and 91 patients (52%) were censored.

**Fig. 1:**
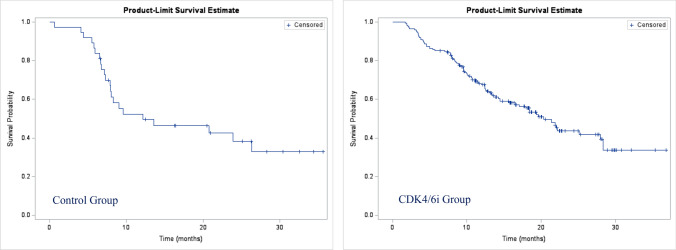
First-line progression-free survival (PFS)

The median time to a progression event or death (PFS) was 12.1 months (95%CI, 7.9–not reached) in the control group vs 20.4 months (95%CI, 15.6–28) in the CDK4/6i group (Fig. 1).

**Fig. 2:**
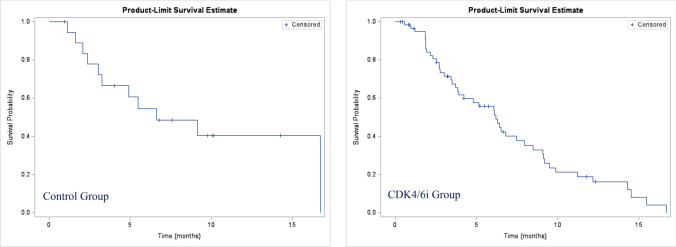
Second-line progression-free survival (PFS2)

The median time for PFS2 was similar, 6.6 months (95%CI 3.0–not reached) in the control group vs 6.2 months (95%CI 4.8–7.9) in CDK4/6i-treated patients (Fig. 2).

In the control group, 11 patients (57.9%) experienced progression events, and 8 patients (42.1%) were censored at the beginning of the study. In the CDK4/6i group, 45 patients (73.8%) experienced an event, and 16 patients (26.2%) were censored at the start of the study..

**Fig. 3:**
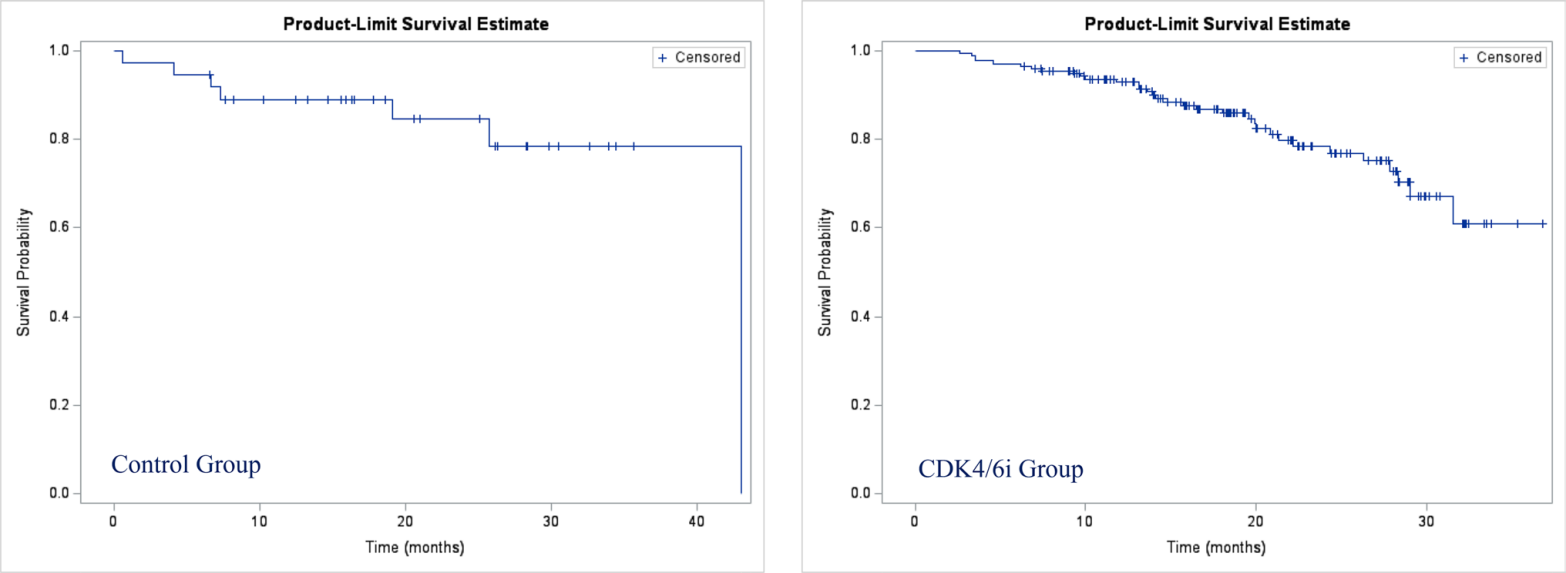
Overall Survival (OS)

The estimated median overall survival (OS) was 43 months (95%CI 31.6–not reached) for the control group and was not reached for the CDK4/6i group (95%CI not reached). For the CDK4/6i group, the overall 12-month survival rate was 92.9% and the 24-month survival rate 78.4% (Fig. 3).

### Safety

Overall, incidence and characteristics of adverse events were very similar for both drugs, except for 8 febrile neutropenia; 5 (3.3%) reported in the Palbociclib group, and 3 (14.3%) in the ribociclib group. AEs are summarized in Table S4; fatigue/asthenia G3/G4 episodes were reported in 9 (6.0%) and 2 (1.3%) palbociclib patients, and in 9 (5.1%) and 1 (4.8%) ribociclib patients, respectively. G3/G4 neutropenia were 68 (45.0%) and 7 (4.6%) in palbociclib patients, and 73 (41.7%) and 0 (0) in ribociclib patients, respectively.

## Discussion

This study was a multicenter retrospective evaluation of the electronic health records of 212 patients with HR + /HER2− advanced breast cancer in Andalusia, 175 (82.5%) patients using CDK4/6 inhibitors and 37 (17.5%) patients using endocrine therapy without CDK4/6 inhibitors (control group) (Figure S1).

Patients´ age has been a conditioning factor when choosing the treatment in clinical practice. In the last few years, there were multiple publications that showed evidence that the treatment should be adjusted to the patient´s comorbidities and prioritize the performance status [18–20]. Indeed, advanced age is classically associated with comorbidity burden, frailty, and lower performance status that limits the use of treatments linked to greater toxicity or greater patient follow-up needs [21–23]. Nevertheless, a recently published Real-World Quality of Life Subanalysis from the POLARIS study [24] showed that in patients over 70 years of age, palbociclib + endocrine therapy did not show any real or meaningful changes or any significant adverse impact on QoL, activities of daily living, or impairment in patients with HR + /HER2–ABC. Similarly, the French PALOMAGE real-life study showed no change in QoL in patients who continued or discontinued palbociclib treatment after 18 months [25]. Additionally, polypharmacy is an area of concern for the elderly as it poses a greater risk of adverse drug reactions. However, pharmacokinetic CDKi reviews [26] and recent recommendations have regarded palbociclib as usually recommended in different patient profiles [25].

In our analysis, we concluded that the treatment was not chosen according to the patients´ ECOG status or comorbidities. When comparing patients´ ECOG 3 status, we found that in the CDK4/6i group, there were 2 (1.4%) patients and 0 in the control group (Table 1). When analyzing the median of age, we found that patients treated with CDKi were in fact younger (58.1 years) than those in the control group (67.6 years) (Table 1). In contrast, other factors, such as KI-67 > 20%, tumour grade 3, and oestrogen negative receptor did not show significant differences between the arms (Table 1).

The time until diagnosis of advanced disease appears to be a clinical factor that affects whether CDK4/6i or hormone therapy alone is prescribed. This may be because de novo patients and those after a long DFI interval have a higher level of hormone sensitivity to endocrine treatments than those who relapse with a shorter DFI interval [27, 28]. In our study, we observed that in de novo patients, the treatment of choice was IA. This is contrary to the P-REALITY and P-REALITY-X studies [14, 29], in which patients with de novo disease were treated in greater proportions with palbociclib plus AI. A consistent benefit of CDK4/6i in combination with AI was observed across all subgroups examined in the P-REALITY and P-REALITY-X studies (older patient groups and among patients with and without visceral metastases or bone-only disease) [14, 29] and also that patients with different comorbidities continued to benefit from palbociclib [30].

Additionally, a higher proportion of patients with multiple lesions (81.1%) appear to be treated with CDK4/6i when compared to oligo-metastatic patients (18.9%) (Table 1). Incorporating CDK4/6i may increase disease control and benefit PFS and OS, as described in the palbociclib trials and studies [28, 31–33]. Visceral lesions (pulmonary and hepatic) are usually an important factor in therapeutic decision making since they are associated with poor prognosis [28]. In our study, there was a tendency to treat patients with visceral metastases with CDK4/6i in a higher proportion, which did not reach significance, possibly because of the low number of patients (Table 1). Although the number of metastatic sites seems to be an important factor in the therapeutic decision, the location did not condition the treatment choice, and in our study, there was no significant differences found.

In RWE publications, a significant benefit was observed in OS and PFS in favour of initiating treatment with palbociclib plus AI when compared to AI in monotherapy in the subgroup analyses [14]. In contrast, in the Sonia trial, the authors conclude that CDK4/6i in first-line treatment does not provide statistically significant, nor clinically meaningful PFS benefit compared to second-line use in women with HR + , HER2− [34].

As stated previously, real-life data are necessary to evaluate real-life effectiveness since the very restrictive inclusion and exclusion criteria of the trials limit generalization to the usual clinical practice population [35]. Notably, in our study in the CDK4/6i group, patients were heavily treated with palbociclib (86%) which effectively means this was the largest driver of the effect seen in this study. Patients benefited greatly of a first-line therapy with CDK4/6i, as shown by a CBR of 82%, a median for PFS twice the value of the control group (20.4 months [95% CI 15.6–28] vs 12.1 [95% CI 7.9–not reached]).

Furthermore, even though patients with anticipated worst prognosis factors were in the CDK4/6i group (younger, time of diagnosis, and multiple lesions), the CBR was high for the first-line treatment (82% vs. 67%). These results are in line with the strong evidence from the PALOMA-2 trial, where the CBR was around 85% among all patients randomised to CDK4/6i plus AI [33], and aligned with other real-life studies [36, 37].

The clinical effectiveness of CDK4/6 inhibitors in survival outcomes observed in this study is complementary to the clinical efficacy findings in randomized clinical trials [30, 33, 38, 39] and consistent with that reported in other RWE studies [14, 16, 40, 37, 41, 42]. Patients showed a similar profile to those treated in other real-world studies, where younger women with a de novo MBC diagnosis and with a high burden of the disease were generally more frequently treated with first-line CDK4/6 inhibitors [14, 29]. For example, in the P-REALITY study (*n* = 1430), the palbociclib + letrozole cohort was younger with a median age (IQR) of 66 (58–73) vs. 70 (61–79) in the letrozole group, and in the P-REALITY-X (*n* = 2888), the median age (IQR) was 67 (61–74) in the palbociclib + aromatase inhibitor (AI) group vs. 72 (64–80) in the aromatase alone group [14, 29]. This patient profile could anticipate unequal access owing to clinical and demographic characteristics that do not justify the lack of benefits for these patients.

As in the study by Goyal [16], in our population, there were no differences in the therapeutic decisions based on the area where the patient was living (rural vs. others). This finding implies that oncologists did not consider that CDKi treatment needs closer or continuous follow-up since patients in rural regions have a limited ability to visit the clinic because of the distance to the urban centre. Patients in the CDK4/6i arm were younger despite living in the rural environment. This finding can be probably related to the greater possibility for these patients to assist to the hospital (Table 1).

In this study, the impact of palbociclib on survival outcomes showed a median PFS of 20 vs. 12 months in the control group. However, the median PFS in the PALOMA-2 trial was significantly longer in the CDK4/6i plus AI group, at 24 months (95% 22.1 to not estimable) [33]. The inherent implications of real-world analyses using data collected during routine care including patients with the worst anticipated prognosis may have contributed to the lower median. Other RWE studies, such as the P-REALITY and P-REALITY-X studies, achieved similar results in terms of PFS: 20 months (95% CI 17.5–21.9) among patients treated with palbociclib plus letrozole and 19.3 months (95% CI 17.5–20.7) in the palbociclib combination group, respectively [14, 29]. Outstandingly, data from the PALBOSPAIN study showed a median PFS greater than 24 months for patients receiving palbociclib + endocrine therapy [43].

In our study, the overall 12-month survival rate was 92.9% and the 24-month survival rate was 78.4% within the range of those published in the EU-IRIS (95% [12 months] and 83% [24 months]), respectively for patients treated with palbociclib plus IA [44]. Likewise, in the P-REALITY study, the CDK4/6i-treated cohort showed a 24-month OS rate of 78.3%, and in the unadjusted Kaplan–Meier analysis of the MEDICARE study, the OS rate at 3 years after treatment initiation was 73.0% for the CDK4/6i plus ET group [16, 29]. Unfortunately, OS data from the PALOMA-2 trial were inconclusive due to the large amount of missing data [33].

The PFS results of the second-line treatment (PFS2) showed that initial treatment with CDK4/6i did not adversely affect subsequent lines of treatment, thereby maintaining the effect achieved by palbociclib in the first-line treatment. This was confirmed by data from the P-REALITY-X study for rwPFS2 and the time to chemotherapy [14, 45], and the time to subsequent therapy and time to chemotherapy for the PALOMA-2 trial [33]. In those studies, rwPFS2 was defined as the number of months from the start of palbociclib + AI or AI alone to disease progression on the second line of therapy, as determined by the treating physician, or death from any cause, whichever occurred first, and time to chemotherapy. TSC (time to subsequent therapy) was defined as the length of time from the start of treatment to the next line of chemotherapy, death from any cause, last visit, or end of study, whichever came first.

Finally, the safety data suggest that CDK4/6i has a similar and favorable safety profile as reported in previous pivotal studies. In addition, in our analysis, safety and tolerability results were good and consistent with expected safety profiles.

This study had some limitations. Due to the retrospective nature of this study cohort, the treatment allocation was not controlled. Thus, the results of this study are potentially affected by several biases, including treatment and channeling biases, precluding control for unmeasured confounders. Another limitation is the small size of the groups, which implies a limitation of statistical power as well as the possible presence of some type of bias. Third, it could be argued that the lack of description of the treatments in the control group can obscure the true effects of the treatment under investigation. Finally, the fact that the patients in the study all came from only one region of Spain may limit the generalizability of the findings to other populations. The healthcare system characteristics and demographic data of the study population may not be representative of the broader population, which makes it difficult to extrapolate the results to other regions or countries.

To the best of our knowledge and despite these limitations, this study adds valuable information to the existing body of real-world evidence on patients with HR + /HER2-MBC who received CDK4/6i in Andalusia, mainly palbociclib, as a highly efficacious, well tolerated, and convenient treatment.

## Conclusions

This is a descriptive study of the patients with RH + /Her2− MBC treated in Andalusia, which explains the clinical and demographic characteristics that influence on the treatment of choice with CDK4/6i for these population groups. Overall, younger patients and with anticipated worst prognostic factors were more frequently treated with CDK4/6i. In our analysis, the characteristics of the patients most frequently treated with CDK4/6i as first-line treatment were younger, had multiple metastatic sites, and the biopsies of the metastatic site at the moment of the relapse were most commonly performed in the Autonomous Community of Andalusia.

RWE studies are always required in clinical studies because they can provide a better understanding of effectiveness, safety, and tolerability of treatment in real-world settings. The current study provides a comprehensive understanding of effectiveness and safety of CDK4/6i treatment in patients with breast cancer from Andalusia. Additionally, this study can provide insights into the challenges and opportunities associated with the practical implementation of CDK4/6i treatment in a clinical setting.

The effectiveness has been demonstrated in terms of clinical benefit and survival outcomes. Therefore, it adds to the growing body of literatures, including clinical trials and real-world data, which supports the use of CDK4/6i and endocrine therapy as a standard of care for patients with HR + /HER2 − .

## Supplementary Information

Below is the link to the electronic supplementary material.

Supplementary file1 (DOCX 23 kb)
