# Supplementary material for: Retrospective registry of patients with locally advanced/metastatic HR+/HER2− breast cancer treated in clinical practice in Andalusia
Source: Clin Transl Oncol. 2024 Jun 3;26(12):3131–41. doi: 10.1007/s12094-024-03510-8 (PMC11564311; doi:10.1007/s12094-024-03510-8)
Supplement: Supplementary file 1 — Supplementary file1 (DOCX 23 kb) [file 12094_2024_3510_MOESM1_ESM.docx]

Supplementary Appendix 1

Figure S1. Flowchart of study patients.

Table S1. Baseline Demographic and Clinical Characteristics of Study Patients according to the treatment line.

|  | **1st line of treatment** | **2nd line of treatment** | **3rd line of treatment** | ***p*-value** |
| --- | --- | --- | --- | --- |
| **Age (years), *n*** | 114 | 29 | 32 | >0.05 |
| *Mean (SD)* | 58.7 (11.5) | 58.7 (11.5) | 55.2 (12.9) |  |
| **Sex*, n*** | 114 | 29 | 32 | >0.05 |
| Male | 1 (0.9) | 0 (0) | 0 (0) |  |
| Female | 113 (99.1) | 29 (100) | 32 (100) |  |
| **Menopause*, n*** | 114 | 29 | 32 | >0.05 |
| Yes*, n (%)* | 81 (71.1) | 22 (75.9) | 20 (62.5) |  |
| No*, n (%)* | 32 (28.1) | 7 (24.1) | 12 (37.5) |  |
| Does not apply*, n (%)* | 1 (0.9) | 0 (0) | 0 (0) |  |
| **Living area*, n*** | 114 | 29 | 32 | >0.05 |
| Rural*, n (%)* | 60 (52.6) | 9 (31.0) | 17 (53.1) |  |
| No rural*, n (%)* | 54 (47.4) | 20 (69.0) | 15 (46.9) |  |
| **ECOG*, n*** | 88 | 24 | 27 | >0.05 |
| 0*, n (%)* | 71 (80.7) | 15 (62.5) | 23 (85.2) |  |
| 1*, n (%)* | 12 (13.6) | 8 (33.3) | 2 (7.4) |  |
| 2*, n (%)* | 3 (3.4) | 1 (4.2) | 2 (7.4) |  |
| 3*, n (%)* | 2 (2.3) | 0 (0) | 0 (0) |  |
| 4*, n (%)* | 0 (0) | 0 (0) | 0 (0) |  |

Table S2. Baseline Demographic and Clinical Characteristics of Study Patients according to metastatic tumor placement.

|  | **Visceral** | **No-visceral** | **Bone** | ***p*-value** |
| --- | --- | --- | --- | --- |
| **Age (years), *n*** | 104 | 35 | 72 | >0.05 |
| *Mean (SD)* | 59.2 (12.9) | 63.1 (12.8) | 58.9 (13.4) |  |
| **Sex*, n*** | 114 | 29 | 32 | >0.05 |
| Male | 1 (1.0) | 0 (0) | 0 (0) |  |
| Female | 103 (99.0) | 35 (100) | 721 (100) |  |
| **Menopause*, n*** | 104 | 35 | 72 | >0.05 |
| Yes*, n (%)* | 70 (67.3) | 28 (80.0) | 52 (72.2) |  |
| No*, n (%)* | 33 (31.7) | 7 (20.0) | 20 (27.8) |  |
| Does not apply*, n (%)* | 1 (1.0) | 0 (0) | 0 (0) |  |
| **Living area*, n*** | 104 | 35 | 72 | >0.05 |
| Rural*, n (%)* | 49 (47.1) | 18 (51.4) | 35 (48.6) |  |
| No rural*, n (%)* | 55 (52.9) | 17 (48.6) | 37 (51.4) |  |
| **ECOG*, n*** | 88 | 24 | 27 | >0.05 |
| 0*, n (%)* | 60 (74.1) | 24 (82.8) | 43 (82.7) |  |
| 1*, n (%)* | 14 (17.3) | 3 (10.3) | 8 (15.4) |  |
| 2*, n (%)* | 5 (6.2) | 2 (6.9) | 1 (1.9) |  |
| 3*, n (%)* | 2 (2.5) | 0 (0) | 0 (0) |  |
| 4*, n (%)* | 0 (0) | 0 (0) | 0 (0) |  |

Table S3**.** Clinical Characteristics of Study Patients according to metastatic tumor placement

|  | **Palbociclib** | **Ribociclib** |
| --- | --- | --- |
| **Number of CDK 4/6i cycles received*, n*** | 151 | 21 |
| *Mean, SD* | 15.0 (9.1) | 22.9 (8.0) |
| **Patients continuing CDK 4/6i treatment at the time of inclusion*, n*** | 151 | 21 |
| Yes, *n (%)* | 72 (47.7) | 15 (71.4) |
| No, *n (%)* | 79 (52.3) | 6 (28.6) |
| **Reason for termination of CDK 4/6i treatment*, n*** | 79 | 6 |
| Progression, *n (%)* | 70 (88.6) | 6 (100) |
| Adverse event, *n (%)* | 7 (8.9) | 0 (0) |
| Other, *n (%)* | 2 (2.5) | 0 (0) |
| **Time on treatment with CDK 4/6i (months)*, n*** | 79 | 6 |
| *Mean, SD* | 9.9 (6.6) | 12.9 (5.7) |
| **Patients with dose reduction of CDK 4/6i*, n*** | 151 | 21 |
| Yes, *n (%)* | 60 (39.7) | 4 (19.0) |
| No, *n (%)* | 91 (60.3) | 17 (81.0) |
| **Number of dose reductions of treatment with CDK 4/6i*, n*** |  |  |
| 1 reduction, *n (%)* | 43 (71.7) | 4 (100) |
| 2 reductions, *n (%)* | 17 (28.3) | 0 (0) |
| 3 reductions, *n (%)* | 0 (0) | 0 (0) |
| **New dosage after dose reduction of treatment with CDK 4/6i*, n*** | 60 | 4 |
| 75 mg, *n (%)* | 18 (30.0) | 0 (0) |
| 100 mg, *n (%)* | 42 (70.0) | 0 (0) |
| 200 mg, *n (%)* | 0 (0) | 0 (0) |
| 400 mg, *n (%)* | 0 (0) | 4 (100.0) |
| **Patients with dose interruption of treatment with CDK 4/6i*, n*** | 151 | 21 |
| Yes, *n (%)* | 82 (54.3) | 6 (28.6) |
| No, *n (%)* | 69 (45.7) | 15 (71.4) |
| **Number of dose interruptions during treatment with CDK 4/6i*, n*** | 82 | 6 |
| 1 interruption, *n (%)* | 26 (31.7) | 4 (66.7) |
| 2 interruptions, *n (%)* | 20 (24.4) | 1 (16.7) |
| 3 interruptions, *n (%)* | 14 (17.1) | 0 (0) |
| 4 interruptions, *n (%)* | 11 (13.4) | 1 (16.7) |
| 5 or more interruptions | 11 (13.4) | 0 (0) |
| **Reason for dose interruption of CDK 4/6i therapy*, n*** | 225 | 10 |
| Adverse event, *n (%)* | 201 (89.3) | 9 (90.0) |
| Other, *n (%)* | 24 (10.7) | 1 (10.0) |
| **Distribution of the duration of treatment discontinuation with CDK 4/6i*, n*** | 226 | 10 |
| < 1 week, *n (%)* | 2 (0.9) | 0 (0) |
| 1 week, *n (%)* | 170 (75.2) | 8 (80.0) |
| 1-2 weeks, *n (%)* | 7 (3.1) | 0 (0) |
| 2 weeks, *n (%)* | 31 (13.7) | 0 (0) |
| 3 weeks, *n (%)* | 4 (1.8) | 0 (0) |
| 4 weeks/1 month, *n (%)* | 9 (4.0) | 1 (10.0) |
| 5 weeks, *n (%)* | 2 (0.9) | 0 (0) |
| 3 months, *n (%)* | 0 (0) | 0 (0) |
| 5 months, *n (%)* | 1 (0.4) | 1 (10.0) |
| **Duration of treatment interruption with CDK 4/6i*, n*** | 226 | 10 |
| *Mean, SD* | 10.1 (10.9) | 17.6 (26.4) |
| **Survival*, n*** | 151 | 21 |
| *Exitus*, *n (%)* | 31 (20.5) | 2 (9.5) |
| Censored, *n (%)* | 120 (79.5) | 19 (90.5) |

Table S4. Safety data of CDK4/6i treatments.

|  | **Palbociclib** | | **Ribociclib** | |
| --- | --- | --- | --- | --- |
| **Febrile neutropenia*, n*** | 151 | | 21 | |
| Yes, *n (%)* | 5 (3.3) | | 3 (14.3) | |
| No, *n (%)* | 146 (96.7) | | 18 (85.7) | |
| **QTc prolongation during treatment*, n*** | 151 | | 21 | |
| Yes, *n (%)* | 0 (0) | | 0 (0) | |
| No, *n (%)* | 25 (16.6) | | 13 (61.9) | |
| Unknown, *n (%)* | 126 (83.4) | | 8 (38.1) | |
| **Adverse events** | **Grade 3** | **Grade 4** | **Grade 3** | **Grade 4** |
| Fatigue/asthenia, *n (%)* | 9 (6.0) | 2 (1.3) | 9 (5.1) | 1 (4.8) |
| Neutropenia, *n (%)* | 68 (45.0) | 7 (4.6) | 73 (41.7) | 0 (0) |
| Increased ALT, *n (%)* | 1 (0.7) | 0 (0) | 2 (1.1) | 0 (0) |
| 🠝ALT + bilirubin, *n (%)* | 1 (0.7) | 0 (0) | 1 (0.6) | 0 (0) |
| Increased AST, *n (%)* | 2 (1.4) | 0 (0) | 3 (1.7) | 0 (0) |
| 🠝AST + bilirubin, *n (%)* | 1 (0.7) | 0 (0) | 1 (0.6) | 0 (0) |

ALT=Alanine transaminase; AST=Aspartate transaminase; QTc= Corrected QT Interval.
